# Repertoire of microRNAs in Epithelial Ovarian Cancer as Determined by Next Generation Sequencing of Small RNA cDNA Libraries

**DOI:** 10.1371/journal.pone.0005311

**Published:** 2009-04-23

**Authors:** Stacia K. Wyman, Rachael K. Parkin, Patrick S. Mitchell, Brian R. Fritz, Kathy O'Briant, Andrew K. Godwin, Nicole Urban, Charles W. Drescher, Beatrice S. Knudsen, Muneesh Tewari

**Affiliations:** 1 Division of Clinical Research, Fred Hutchinson Cancer Research Center, Seattle, Washington, United States of America; 2 Division of Human Biology, Fred Hutchinson Cancer Research Center, Seattle, Washington, United States of America; 3 Division of Public Health Sciences, Fred Hutchinson Cancer Research Center, Seattle, Washington, United States of America; 4 Department of Medical Oncology, Fox Chase Cancer Center, Philadelphia, Pennsylvania, United States of America; Cincinnati Children's Research Foundation, United States of America

## Abstract

**Background:**

MicroRNAs (miRNAs) are small regulatory RNAs that are implicated in cancer pathogenesis and have recently shown promise as blood-based biomarkers for cancer detection. Epithelial ovarian cancer is a deadly disease for which improved outcomes could be achieved by successful early detection and enhanced understanding of molecular pathogenesis that leads to improved therapies. A critical step toward these goals is to establish a comprehensive view of miRNAs expressed in epithelial ovarian cancer tissues as well as in normal ovarian surface epithelial cells.

**Methodology:**

We used massively parallel pyrosequencing (i.e., “454 sequencing”) to discover and characterize novel and known miRNAs expressed in primary cultures of normal human ovarian surface epithelium (HOSE) and in tissue from three of the most common histotypes of ovarian cancer. Deep sequencing of small RNA cDNA libraries derived from normal HOSE and ovarian cancer samples yielded a total of 738,710 high-quality sequence reads, generating comprehensive digital profiles of miRNA expression. Expression profiles for 498 previously annotated miRNAs were delineated and we discovered six novel miRNAs and 39 candidate miRNAs. A set of 124 miRNAs was differentially expressed in normal versus cancer samples and 38 miRNAs were differentially expressed across histologic subtypes of ovarian cancer. Taqman qRT-PCR performed on a subset of miRNAs confirmed results of the sequencing-based study.

**Conclusions:**

This report expands the body of miRNAs known to be expressed in epithelial ovarian cancer and provides a useful resource for future studies of the role of miRNAs in the pathogenesis and early detection of ovarian cancer.

## Introduction

Epithelial ovarian cancer is the leading cause of gynecologic cancer-related deaths in the United States [1], with late-stage diagnoses having a <30% five-year survival rate [2]. Survival rates could be improved by a better understanding of molecular pathogenesis, which may lead to development of superior targeted therapies, as well as by earlier detection of disease at a surgically curable stage. When detected at a stage in which disease is confined to the ovary, for example, the five-year survival rate increases to >80%. Clinically effective biomarkers for early detection of ovarian cancer could substantially improve survival rates and ovarian cancer biomarker discovery is an important area of ongoing research [3].

MicroRNAs (miRNAs) are a class of small (∼22 nt) non-coding RNA molecules that act post-transcriptionally to regulate gene expression [4]. MicroRNAs originate from hairpin RNA precursors that are processed to generate both the functional mature miRNA and a miRNA “star form” of similar length derived from the opposite strand of the hairpin. MicroRNA-mediated modulation of biological systems has been found to be perturbed in multiple diseases [5], including cancer [6]–[8]. Expression patterns of miRNAs correlate with tissue of origin [8], [9], prognosis [10], [11] and with clinical cancer behaviors [12], making miRNAs valuable tissue-based biomarkers. Furthermore, we and others have recently shown that tumor-derived miRNAs enter the bloodstream at measurable levels, indicating that miRNAs released by tumor tissue represent a powerful new class of blood-based, minimally invasive biomarkers for cancer detection [13]–[16]. As such, there is a strong impetus for a comprehensive analysis of the miRNA repertoire expressed in epithelial ovarian cancer.

Several recent reports have begun to characterize miRNA expression in ovarian cancer using microarrays spotted with probes for a varying number of known miRNAs [17]–[20]. Although these are pioneering studies, they also have limitations. The foremost of these is that all arrays reported to date have incomplete coverage of known miRNAs. Of the 959 miRNAs and star forms present in miRBase (release 12.0) [21], [22], most microarray platforms applied to date assay only a few hundred miRNAs, with the most comprehensive of the arrays assaying 470 miRNAs and star forms. In addition, microarray approaches only measure previously identified miRNAs and are not equipped to discover and profile novel miRNAs. This is an important distinction to make given that several reports suggest that a significant number of miRNAs, particularly those that may be cell-type specific in expression, remain to be discovered [23], [24]. Finally, because of the small size of miRNAs and the challenges of achieving uniform hybridization conditions across an entire miRNA microarray, there is potential for cross-hybridization of miRNAs that are highly related in sequence, making it sometimes impossible to definitively distinguish between miRNAs differing by one or two nucleotides.

To overcome the limitations associated with microarray studies, we used “next generation” sequencing technology (specifically massively parallel pyrosequencing using the 454 Life Sciences platform) of small RNA cDNA libraries to more comprehensively characterize miRNA expression in epithelial ovarian cancer, as well as in primary cultures of normal ovarian surface epithelial cells. This approach, which is based on 5′ and 3′ universal linker ligation/RT-PCR and counting of sequence reads obtained for distinct miRNAs, can in theory capture all miRNAs present in the RNA samples (including ones of novel sequence) and permits precise identification of miRNAs, even when differing by only a single nucleotide. We generated a total of 738,710 sequence reads from four small RNA cDNA libraries representing normal primary human ovarian surface epithelium (HOSE) cultures and three common epithelial ovarian cancer subtypes (serous, clear cell and endometrioid). Here we describe the results of this effort, including the identification and profiling of both previously annotated and novel miRNAs expressed in ovarian cancer.

## Methods

Additional details regarding cell culture, RNA isolation, small RNA cDNA library construction, massively parallel sequencing, computational pipeline description and miRNA TaqMan quantitative reverse-transcription polymerase chain reaction (qRT-PCR) are provided in detail in **Supplementary** **Methods S1** and in [23].

### Cell culture and clinical materials

Normal primary human ovarian surface epithelial (HOSE) cells were obtained from the normal ovaries of postmenopausal women using a modification of the technique described previously in [25]. In all cases, specimens were taken from normal-appearing ovarian surface epithelium, which was confirmed following histopathological review. HOSE specimens were obtained under a protocol that was approved by the Research Review Committee and Internal Review Board at the Fox Chase Cancer Center from patients undergoing clinically indicated surgery (either prophylactic risk reduction salpingo-oophorectomy or total abdominal hysterectomy with bilateral salpingo-oopherectomy) and who provided written informed consent for tissue not required for diagnosis to be used for research. Primary HOSE cells were cultured using media described in [26] at 37°C in 5% CO_2_.

Ovarian cancer snap-frozen specimens corresponding to Stage III/IV epithelial ovarian cancer (19 serous, four clear cell and 10 endometrioid) contained >70% malignant epithelial cellularity as assessed by pathologist review of an adjacent stained tissue section. Ovarian cancer specimens were obtained from the Pacific Ovarian Cancer Research Consortium Repository. Specimens were collected under a protocol approved by the Fred Hutchinson Cancer Research Center Institutional Review Board and were obtained from patients who had provided written informed consent for tissue not required for clinical management purposes to be used for research.

### RNA extraction and miRNA library preparation

Normal primary HOSE cultures (passage 4–7) were lysed in 600 µl mirVana Lysis/Binding buffer (Ambion, Inc., Austin, TX). Tumor specimens (∼100 mg each) were homogenized in a Qiagen Tissuelyser with 600 µl mirVana Lysis/Binding buffer. Total RNA was extracted from both sample types using the mirVana™ miRNA isolation kit (Ambion) per the manufacturer's protocol. RNA from snap-frozen tumors (19 serous, four clear cell and 10 endometrioid tumors, respectively) was pooled according to histologic subtype. MicroRNA cloning was performed as previously described [27], (http://web.wi.mit.edu/bartel/pub/protocols/miRNACloningUpdate0705.pdf) with modifications at the amplification steps to prepare the samples for massively parallel sequencing by 454 Life Sciences.

### Custom bioinformatic data analysis pipeline

Processing and annotation of sequences based on identity to known transcribed RNAs or as novel miRNAs was performed using a custom bioinformatics pipeline described in detail in **Supplementary** **Methods S1**.

### Measurement of miRNA levels in RNA from clinical samples using TaqMan® qRT-PCR assays

Total RNA was reverse transcribed and prepared for qRT-PCR using the TaqMan® miRNA Reverse Transcription Kit and miRNA-specific stem-loop primers (Applied BioSystems, Inc.) as previously described [23]. Data was analyzed with SDS Relative Quantification Software version 2.2.2 (Applied BioSystems, Inc.), with the automatic Ct setting for assigning baseline and threshold for Ct determination.

## Results

### Overview of small RNA cDNA libraries and 454 sequencing

**Figure 1:**
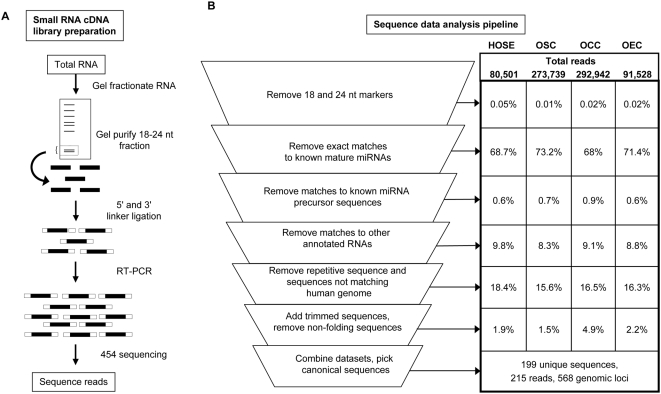
Small RNA cDNA library generation and data analysis pipeline. *A*. Small RNAs were isolated from normal primary HOSE cultures and from serous (OSC), clear cell (OCC) and endometrioid (OEC) ovarian cancer tissues. Following 5′ and 3′ linker ligation, RT-PCR was performed to generate four independent cDNA libraries of small RNAs that were then used as templates for massively parallel pyrosequencing (454 sequencing). *B*. Description of the steps in the data analysis pipeline. The initial step of the data analysis was removal of sequences corresponding to 18 nt and 24 nt RNA markers that had been spiked into the total RNA prior to gel electrophoresis. Percentage of total reads from HOSE, OSC, OCC and OEC datasets classified into the designated categories and filtered out at each step are listed in the table on the right. At the bottom of the table, the number of “unique sequences” represents the non-redundant sequences derived after collapsing multiple reads of identical sequence. Some canonical sequences map to more than one locus in the genome. At completion of the analysis, 199 sequences totaling 215 reads and mapping to 568 loci met our criteria for canonical hairpin-derived sequences from the combined datasets.

Small RNA cDNA libraries were constructed from RNA sample pools isolated from primary cultures of histologically normal primary HOSE (*n* = 4) and epithelial ovarian cancer specimens of serous (OSC, *n* = 19), clear cell (OCC, *n* = 4) and endometrioid (OEC, *n* = 10) histologies. Tumor specimens were selected to contain at least 70% malignant epithelial cells. Total RNA was size-fractionated via polyacrylamide gel electrophoresis and small RNAs corresponding in molecular weight to the mature miRNA population (18–24 nt fraction) were gel extracted and processed for reverse transcription and PCR amplification to create cDNA libraries (**Figure 1A**). Massively parallel pyrosequencing using the 454 Life Sciences' platform generated 80,501 normal HOSE reads, 273,739 OSC reads, 292,942 OCC reads, and 91,528 OEC reads (**Figure 1B**). These corresponded to 10,544 HOSE, 26,849 OSC, 37,792 OCC and 13,134 OEC non-redundant sequences. The greater number of reads corresponding to serous and clear cell samples was due to greater depth of sequencing (i.e., a larger portion of the sequencing plate used) rather than to any particular variations during library preparation.

Sequence data was processed using our custom computational pipeline **(** **Figures 1B** **,** **S1** **and** **Table S1** **)**. The initial steps of the pipeline identified sequence matches to databases of previously annotated RNAs (e.g., known miRNAs, other noncoding RNAs, messenger RNAs) and to highly repetitive sequence elements. Remaining sequences were processed to identify novel miRNAs (**Figure S1**; details of the computational pipeline are provided in **Supplementary** **Methods S1**). In the next sections, we discuss in detail the sequences matching to known miRNAs and the identification of novel miRNAs.

#### MicroRNA profiling: previously annotated miRNAs

**Figure 2:**
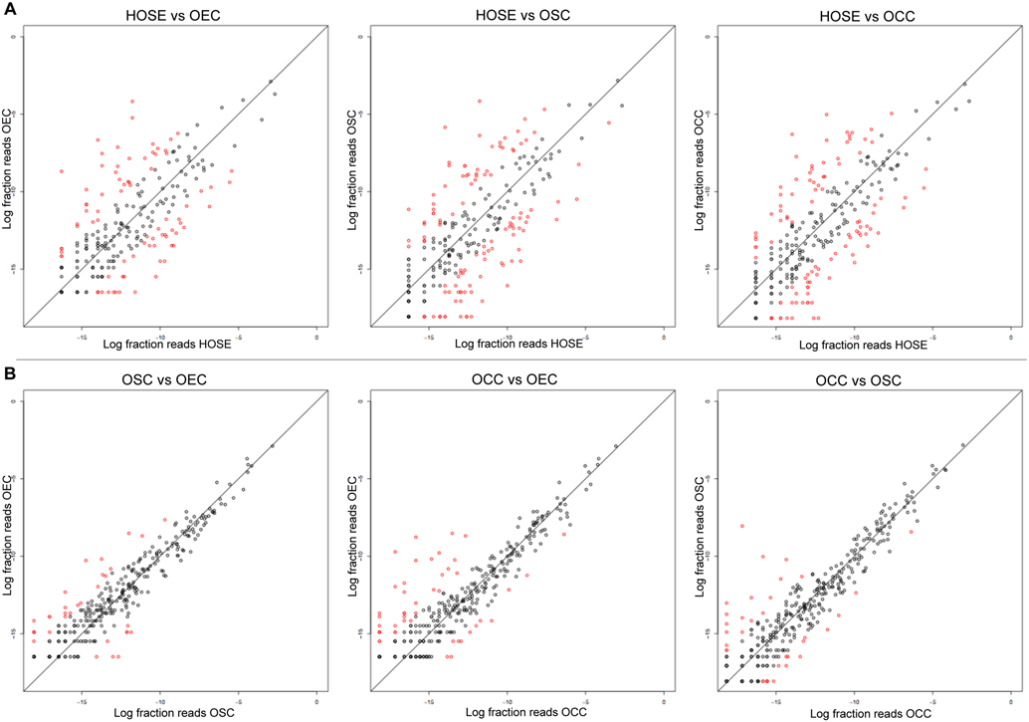
Comparison of abundance of known miRNAs across datasets. Datasets are compared pairwise, plotting the log of the fraction of total reads for a given miRNA in a given dataset against its corresponding value in the second dataset. For each plot, only miRNAs that were sequenced in both datasets are plotted; miRNAs that were sequenced only in one of the two datasets are not shown. The top row (*A*), compares ovarian cancer datasets (OSC, OCC and OEC) to normal primary HOSE cultures; the bottom row (*B*), compares ovarian tumor histologic subtypes to each other. Red dots signify miRNAs that had a fold change ≥4.

Matches to known miRNAs in miRBase (release 12.0) [21], [22] represented 68–73% of the reads, depending on the dataset (**Figure 1B** **and** **Table S1**) and corresponded to a total of 498 known miRNAs (including star forms). The cloning frequency of individual miRNAs, expressed as a fraction of total reads obtained from a given sample, can be used to compare relative expression of miRNAs between samples [28]–[30]. The global summary of our 454 sequencing datasets is presented in **Figure 2**, which depicts the relative expression of miRNAs between normal primary HOSE and cancer tissue datasets (*A*), as well as between the three epithelial ovarian subtypes (*B*). The most differentially expressed miRNAs (i.e., ≥4 fold-change) are shown as red dots. It is notable, though not unexpected, that the ovarian cancer histologic subtype miRNA profiles were more different from normal HOSE (*A*) than they were from each other (*B*). Tabulated 454 sequencing data for miRNA abundance in all datasets, as well as miRNA names and abundance ratios for all pairwise comparisons between sample types, are provided in their entirety in **Table S2**.

**Figure 3:**
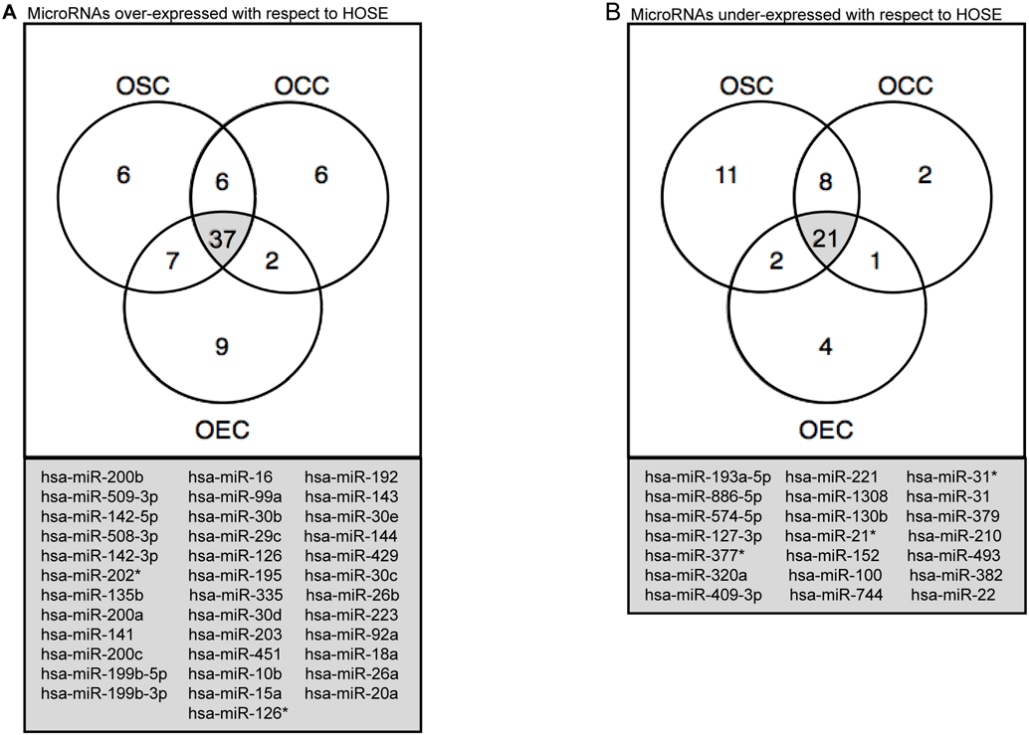
Differential expression of miRNAs in ovarian tumor histologic subtypes relative to normal primary HOSE. Venn diagrams depict numbers of miRNAs differentially expressed in ovarian cancer histologic subtypes relative to normal primary human ovarian surface epithelium (HOSE) cultures. Criteria for a miRNA as differentially expressed was defined by a fold-change ≥4 with a Fisher's exact test *P*-value<5.0×10^−6^ for miRNAs that had detectable expression in both HOSE and ovarian cancer samples, or by a Fisher's exact test *P*-value<5.0×10^−6^ alone in cases where a fold-change value was indefinable due to zero reads for a given miRNA in either the HOSE or ovarian cancer comparison sample. A total of 74 over-expressed miRNAs (*Panel A*) and 49 under-expressed miRNAs (*Panel B*) in ovarian cancer with respect to normal HOSE were identified. MicroRNAs that were found to be consistently over-expressed or consistently under-expressed in all three ovarian cancer histologic subtypes are listed by name.

The overlap of differentially expressed miRNAs between subtypes is visualized in the Venn diagrams in **Figure 3**, which also incorporate miRNAs excluded from **Figure 2** because of zero reads in either normal HOSE or ovarian cancer samples. From the intersection of the three groups in the Venn diagrams, it is evident that in many cases miRNAs differentially expressed in ovarian cancer relative to normal HOSE show the same pattern of differential expression regardless of histologic subtype. **Table S3** provides a full list of names of individual miRNAs and expression ratio and *P*-values for the comparisons summarized in **Figure 3**.

We directly compared miRNA expression between ovarian cancer histologic subtypes by deriving expression ratios for each miRNA based on fractional abundance values. Significance was assessed using Fisher's exact test. The top ten miRNAs differentially expressed in each of the pairwise comparisons of ovarian cancer histologic subtypes are listed in **Table S4** (results for all miRNAs are provided in their entirety in **Table S2**). Among the most histologic subtype-specific miRNAs are miR-449a (serous-specific), miR-499-5p/miR-375/miR196a/miR-196b/miR-182 (endometrioid-specific) and miR-486-5p/miR-144/miR-30a/miR-199a-5p (clear cell-specific).

#### Validation of 454 sequencing miRNA expression results by qRT-PCR

**Figure 4:**
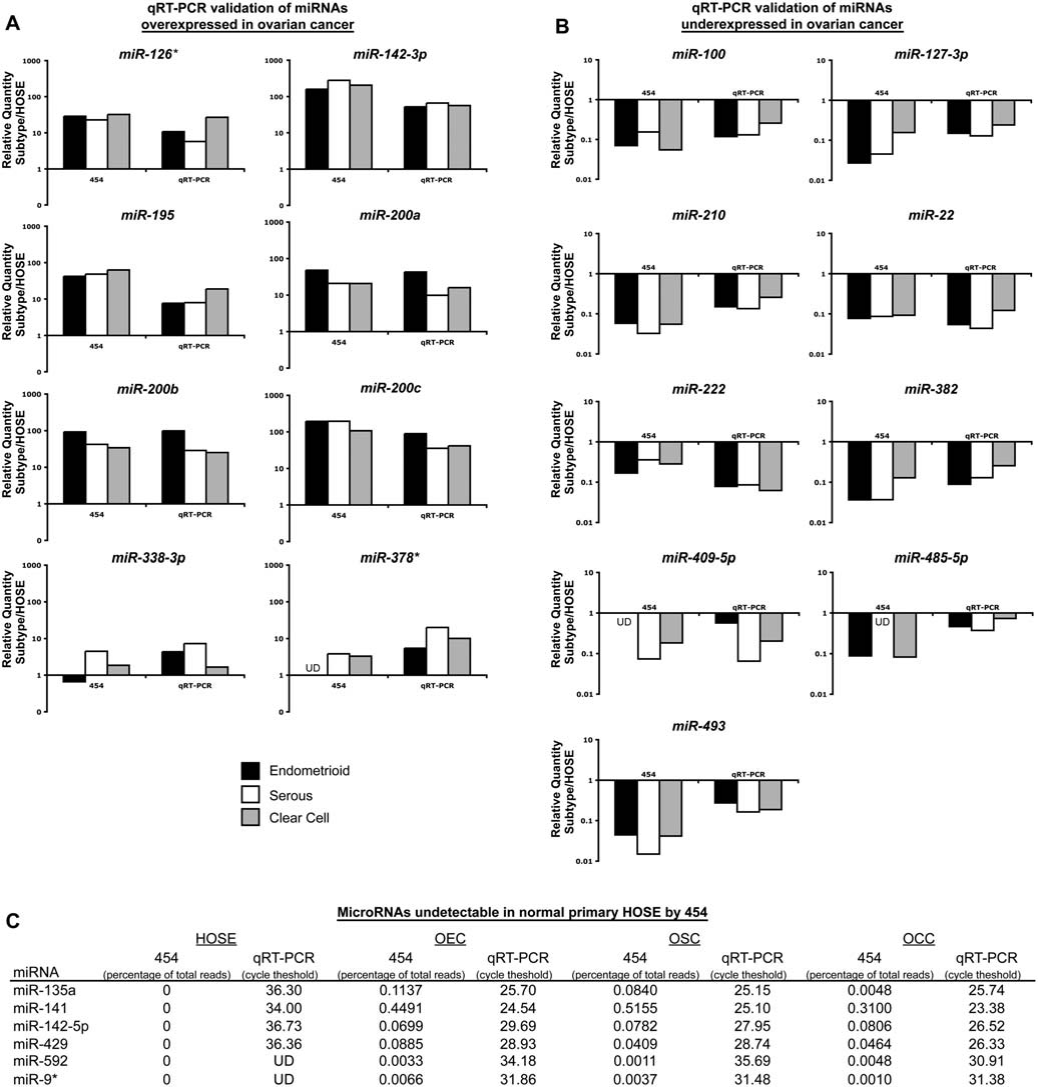
Validation of 454 sequencing-derived miRNA expression results by qRT-PCR: miRNAs differentially expressed in ovarian cancer relative to normal HOSE. The graphs display miRNA Taqman qRT-PCR results for a sampling of miRNAs identified by 454 sequencing to be over-expressed (*Panel A*) or under-expressed (*Panel B*) in ovarian cancer relative to normal HOSE. Relative expression values derived from 454 sequencing are displayed on the left segment of each graph for comparison. Relative expression values of miRNAs in endometrioid (OEC, black bars), serous (OSC, white bars) and clear cell (OCC, grey bars) cancers are depicted. MicroRNAs that were not detected in normal HOSE by 454 sequencing but were detected in ovarian cancer are presented in *Panel C*, where miRNAs are ordered by descending expression (which corresponds to ascending cycle threshold, Ct) in normal HOSE. 454 sequencing data is given in terms of percentage of total reads within each sample. UD, undetectable by miRNA TaqMan qRT-PCR.

We used commercially available miRNA TaqMan qRT-PCR assays as an independent confirmation of miRNA differential expression identified by 454 sequencing. MicroRNA TaqMan qRT-PCR assays were performed for a sample of 38 known miRNAs that were selected based on over- or under-expression with respect to primary normal HOSE, or based on differential expression between different subtypes of ovarian cancer (calculated based on 454 sequencing data). Taqman assays were carried out on aliquots of the same RNA pools used for sequencing. Thirty-six of the 38 differentially expressed miRNAs examined by TaqMan qRT-PCR demonstrated consistent results with those obtained by 454 sequencing **(** **Figures 4** **and** **5, A, B, C**). Of the eight miRNAs identified by 454 sequencing to be over-expressed in ovarian cancer relative to the normal HOSE group (**Figure 4A**), all eight demonstrated over-expression in serous and clear cell cancer subtypes when measured using TaqMan qRT-PCR, while six of the eight showed consistent results in the endometrioid cancer subtype (miRs-126*,-142-3p, 195, 200a, 200b, 200c, 338-3p, 378*). Nine miRNAs were under-expressed in ovarian cancer relative to normal HOSE (**Figure 4B**), with all subtypes showing consistent results when measured by TaqMan qRT-PCR. There were six miRNAs that were undetectable (zero reads) in normal HOSE cultures (**Figure 4C**), but had detectable expression in OEC, OSC and/or OCC in the 454 sequencing data, and they likewise showed increased expression in ovarian histologic subtype samples versus normal primary HOSE when examined by TaqMan qRT-PCR (**Figure 4C**).

**Figure 5:**
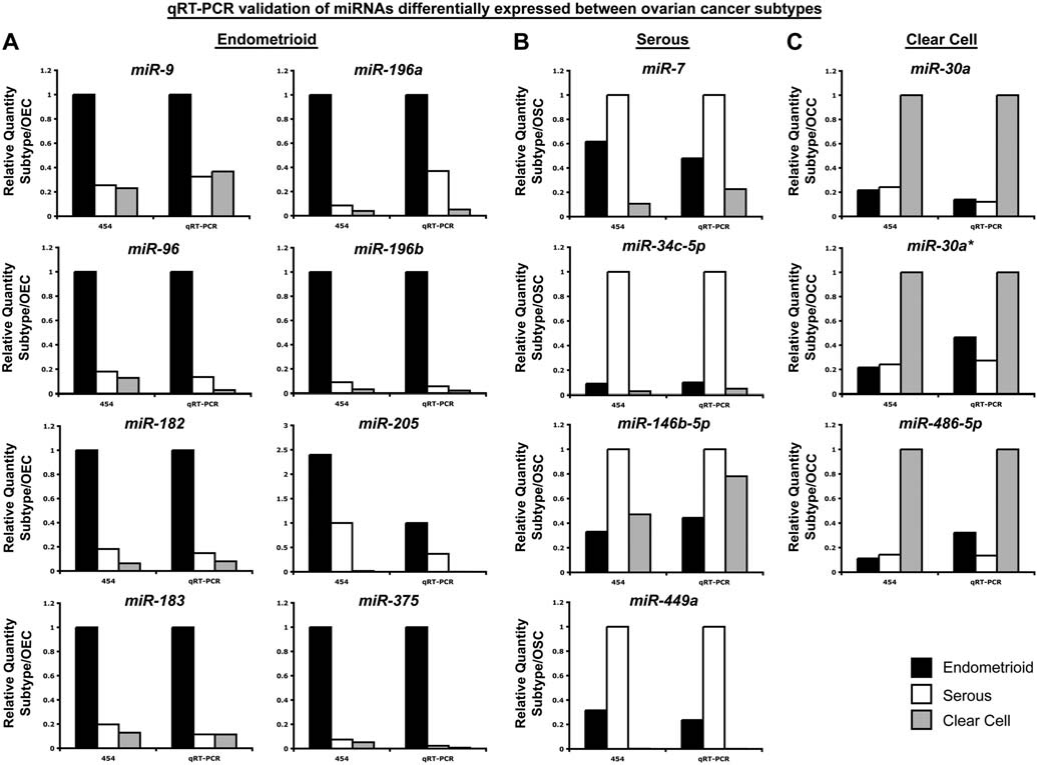
Validation of 454 sequencing-derived miRNA expression results by qRT-PCR: miRNAs differentially expressed between ovarian cancer histologic subtypes. The graphs display Taqman qRT-PCR results for miRNAs differentially expressed between ovarian cancer subtypes, with 454 sequencing-derived data presented for comparison in the left segment of each graph. Relative expression values of miRNAs specifically over-expressed in endometrioid (*Panel A*, OEC, black bars), serous (*Panel B*, OSC, white bars) and clear cell (*Panel C*, OCC, grey bars) cancers are depicted. UD, undetectable by miRNA TaqMan qRT-PCR.

Fifteen miRNAs identified by 454 sequencing to be differentially expressed across ovarian cancer subtypes (**Figure 5A, B, C**) showed similar expression patterns when quantitated by qRT-PCR. Eight miRNAs were over-expressed specifically in endometrioid ovarian cancer (**Figure 5A**), four in serous cancer (**Figure 5B**), and three in clear cell cancer (**Figure 5C**) relative to the other subtypes.

#### Analysis of sequencing data to identify novel hairpin-derived small RNAs

Having validated 454 sequencing results corresponding to differential expression of previously annotated miRNAs, we next turned to the identification of novel miRNA sequences. After filtering out matches to previously annotated features including messenger RNAs, tRNAs and other known noncoding RNAs, the remaining sequences were aligned to the reference human genome sequence (NCBI Build 36.1) [31]. Sequences were required to match the human genome sequence perfectly to be carried further for additional computational analysis, with the only exception to this being sequences that demonstrated addition of 1–3 non-templated nucleotides at the 3′ terminus. In these cases, we trimmed non-templated bases from the original sequence and it was then carried further for analysis [32], [33].

The data processing steps described thus far yielded 841 normal HOSE, 2,840 OSC, 11,224 OCC, and 1,764 OEC unique sequences corresponding to novel small RNAs. These unique sequences corresponded to 1,766 normal HOSE, 5,795 OSC, 19,464 OCC and 3,301 OEC genomic loci that could potentially generate these small RNAs (**Figure 1B** **,** **Table S1**). The data analysis pipeline next screened these genomic loci (in addition to 100 nucleotides of up- and down-stream flanking sequence) for the presence of a predicted hairpin secondary structure by computing free energy, shape probability (as determined by the RNAshapes program [34]) and the Randfold-computed [35] *P*-value of predicted secondary structures. A detailed description of hairpin folding criteria is provided in **Supplementary** **Methods S1** and in [23]. This resulted in 224 normal HOSE, 511 OSC, 500 OCC, and 247 OEC novel small RNAs found to be potentially derived from a precursor hairpin structure.

Sequences passing the above filtering criteria from the four datasets were then combined and sorted with respect to chromosomal coordinates into groups sharing 5′ ends. From each of these groups we chose a “canonical” sequence representing that genomic locus. The canonical sequences were chosen based on a common 5′ terminus, abundance, and sequence length as described previously [23] (additional details are provided in **Supplementary** **Methods S1**). This process further refined the data into a combined list of 199 unique sequences (potentially derived from 568 genomic loci) that we designated as “novel hairpin-derived small RNAs” from which we identified novel and candidate miRNAs.

#### Identifying novel and candidate miRNAs

In order to determine which sequences to designate as novel miRNAs, we screened the set of novel hairpin-derived small RNAs using criteria similar to those used in other recent miRNA discovery studies [23], [33], [36]: *i*. pairing characteristics of the hairpin, *ii*. the presence of multiple reads sharing the same 5′ terminus, *iii*. absence of annotation indicating non-miRNA biogenesis, *iv*. shared seed region with a known animal miRNA and *v*. presence of corresponding miRNA star form read(s). We considered including phylogenetic sequence conservation as a criterion; however, this did not add significantly to our analysis as none of the novel hairpin-derived miRNAs were conserved beyond primates. This is not unexpected, given that most evolutionarily conserved microRNAs are likely to have already been discovered using computational approaches followed up by directed validation experiments.

**Figure 6:**
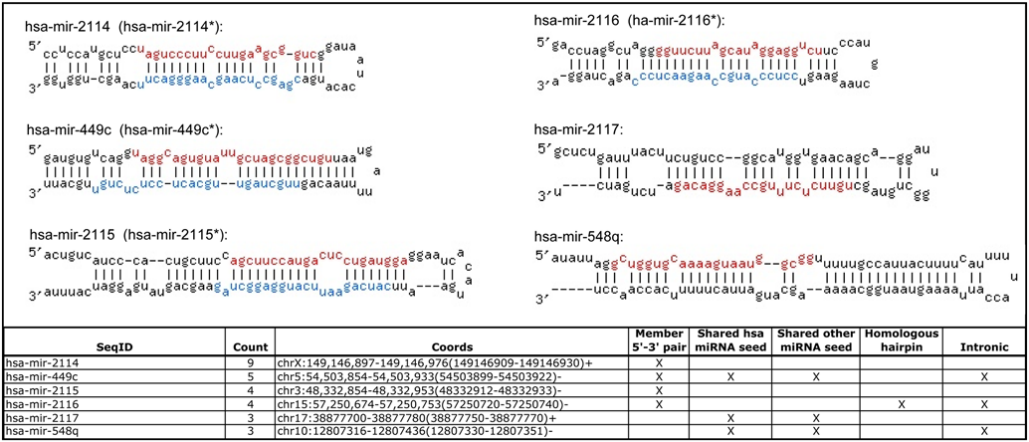
Novel miRNAs discovered by 454 sequencing. Putative secondary structures for the six novel miRNAs discovered in this study are shown. Novel miRNA sequences are shown in red and star forms of novel miRNAs are shown in blue (if identified in our sequencing data). Sequences are novel with respect to miRBase release 12.0 [21], [22]. Identifiers in parentheses refer to the miRNA star forms where these were identified in the 454 sequencing data. Corresponding details of the novel miRNAs are given in the table below the hairpin structures.

Using the criteria above, we identified six novel miRNAs. All of the six met criteria *i*–*iii* and three of the six met criterion *iv* and three met criterion *v* (**Figure 6**; more detail is provided in **Table S5**). In addition, mapping the novel miRNA sequences to the reference human genome sequence revealed that three of the six novel miRNAs are present in introns of other genes (and encoded on the same strand as the respective host genes) (**Figure 6**), much like many previously annotated miRNAs [37], [38]. Two of our novel miRNAs are located in annotated repeats, but have strong enough supporting evidence that they were designated as novel. The context of the predicted precursor structure and the sequence of miRNA star forms corresponding to novel miRNAs is given in **Figure 6**.

The remaining sequences comprised 181 hairpin-derived small RNAs (corresponding to 550 genomic loci). We sought to select from this list the most promising sequences to designate as “candidate miRNAs” that might be confirmed in the future as *bona fide* miRNAs as additional evidence accumulates. Candidate miRNAs were required to meet criteria *i–iii* and have some additional form of supporting evidence (i.e., shared seed region with a known miRNA). This allowed us to refine a final list of 39 candidate miRNAs (potentially originating from 50 genomic loci) (**Table S5**).

## Discussion

The work reported here was motivated by the hypothesis that the entire repertoire of miRNAs expressed in ovarian cancer, including potentially tissue- and cancer-specific miRNAs, had not yet been elucidated. The massively parallel sequencing approach allowed us to be comprehensive (i.e., identifying not only known but also novel miRNAs), and the “digital” nature of the data permitted a semi-quantitative estimation of the relative expression level for many miRNAs. Using this approach, we discovered six novel and 39 candidate miRNAs, and delineated the expression pattern of 498 previously annotated miRNAs in normal primary HOSE cultures and epithelial ovarian cancer tissues. It is notable that of the four published miRNA microarray studies of ovarian cancer [17]–[20], even the most comprehensive of the array platforms was limited to 470 human miRNAs and star forms [17], while the current release of miRBase (release 12.0) [21], [22] contains 969 human miRNAs and star forms. Furthermore, 223 of the known miRNAs we identified and characterized in our sequencing datasets were not represented on even the most comprehensive microarray. Therefore, the results presented here substantially extend the breadth of knowledge of miRNAs expressed in ovarian cancer.

The identification of novel miRNAs in our data is particularly notable. Given that these novel miRNAs have not been detected in multiple large-scale miRNA sequencing studies of other tissues, we speculate that they are expressed in an ovarian cancer-specific manner and may be of special interest as cancer biomarkers. Future studies will be needed to test this hypothesis, as well as to investigate the biological roles of these novel miRNAs in ovarian cancer pathogenesis.

Taken together, the known and novel miRNAs identified in this study provide a pool of candidate miRNA markers for future analysis as blood-based markers for the detection of ovarian cancer. Differential expression between normal and malignant states may provide one method for prioritization of candidates moving forward. We also envision that the miRNAs that we found to be differentially expressed between histologic cancer subtypes could serve a dual purpose as blood-based markers for both cancer detection and histologic classification.

One of the unique features of our study is the use of primary cultures of normal human ovarian surface epithelium as the normal comparison group. Although the ovarian surface epithelium (along with nearby distal fallopian tube epithelium) is considered the origin of epithelial ovarian cancer [39] most other miRNA profiling studies have typically used whole ovary as the normal comparison. This is problematic for identifying miRNAs that are differentially expressed in ovarian cancer relative to normal because the single cell-thick surface epithelial layer comprises far less than 1% of the cellular content of the whole ovary. Performing comparisons of ovarian cancer tissue to an appropriate normal control is a challenging problem in ovarian cancer research. Our approach, although it avoids the problems associated with using whole ovary as a normal sample, has the limitation that we do not know whether and which changes in microRNA expression may be attributed to culture conditions used for primary HOSE cell culture. Future studies incorporating parallel analyses of primary cell cultures derived from ovarian cancer tissues may address this question.

Although the use of different types of “normal” ovarian specimens makes comparison of our data with other studies difficult in most cases, in one microarray study, however, the normal control was human ovarian surface epithelial cells that had been immortalized using a retrovirus expressing human papillomavirus-derived viral oncoproteins E6 and E7 [17], [40]. Because the normal primary HOSE cultures we studied are not immortalized, comparison of our results to that report could identify miRNAs that may be induced by immortalization by E6 and E7 proteins. A notable finding from this comparison is that whereas we identified the miR-200 family of miRNAs (miR-200a/b/c, miR-141 and miR-429) to be expressed at low levels in normal HOSE with highly increased expression in ovarian cancer, the study using E6/E7-immortalized HOSE did not find increased expression of miR-200 family miRNAs in ovarian cancer. This family of miRNAs has important functions in repressing the proteins ZEB1 and ZEB2 [41]–[46], which are transcription factors that function to promote epithelial-to-mesenchymal transition [47]–[51]. One appealing interpretation of the differing results is that primary normal HOSE express low levels of the miR-200 family miRNAs, but the E6 and E7 viral oncoproteins induce miR-200 family expression, which may be an important step in ovarian carcinogenesis given that this miRNA family was the most highly over-expressed miRNA family in all ovarian cancer types we examined. Future work will be needed to address this hypothesis.

In summary, our work expands the current view of the repertoire of miRNAs expressed in normal HOSE and common histologic subtypes of ovarian cancer. Our results represent a resource for future investigations of the role of miRNAs in ovarian cancer and of their applications as clinically useful biomarkers.

The data discussed in this publication have been deposited in NCBI's Gene Expression Omnibus [52] and are accessible through GEO Series accession number GSE15190 (http://www.ncbi.nlm.nih.gov/geo/query/acc.cgi?acc=GSE15190).

## Supporting Information

Table S1 Overview of computational pipeline data processing results. Shaded lines represent steps at which sequences are pruned out of the dataset before the next step. The left column for each dataset contains the number of nonredundant unique sequences, and the right column is the sum of all the reads for each sequence.
(0.02 MB XLS)

Table S2 Identity, abundance data and pairwise relative expression data for all known miRNAs in 454 sequencing datasets. Columns B-I display the number of reads and fractional abundance values for miRNAs corresponding to the 4 sample types studied. Columns J-U provide pairwise comparisons of expression (along with Fisher's exact test P-values) of each miRNA between all combinations of the sample types studied.
(0.21 MB XLS)

Table S3 MicroRNAs differentially expressed between ovarian cancer and normal HOSE.
(0.05 MB XLS)

Table S4 MicroRNAs differentially expressed between ovarian cancer histologic subtypes. Bold blocks of numbers show top 10 most differentially expressed known miRNAs between ovarian cancer subtypes.
(0.04 MB XLS)

Table S5 Novel and candidate miRNAs. Alternating yellow and orange miRNAs represent 5′-3′ pairs, with the yellow indicating the dominant sequence. Pink rows are the two remaining novel miRNAs. The non-highlighted sequences represent candidate miRNAs.
(0.04 MB XLS)

Methods S1 (0.12 MB DOC)

Figure S1 Flow chart of sequence data analysis pipeline. The flow chart shows the steps in the computational analysis of the 454 sequencing data. At each step, sequences may be removed for further analysis, or carried on to the next step in the pipeline. The first steps remove previously annotated features from the pipeline, and then remaining sequences are tested for presence of hairpin secondary structure and other criteria to be designated novel miRNAs. Bolded lower-case letters are referred to in the Supplementary Methods.
(0.77 MB TIF)
