# Supplementary material for: Repertoire of microRNAs in Epithelial Ovarian Cancer as Determined by Next Generation Sequencing of Small RNA cDNA Libraries
Source: PLoS One. 2009 Apr 23;4(4):e5311. doi: 10.1371/journal.pone.0005311 (PMC2668797; doi:10.1371/journal.pone.0005311)
Supplement: Methods S1 — (0.12 MB DOC) [file pone.0005311.s006.doc]

**Supplementary Methods S1**

**Cell Culture and Clinical Materials**

Normal primary human ovarian surface epithelial (HOSE) cells were obtained from the normal ovaries of women using a modification of the technique described previously in [1]. Samples were collected from four post-menopausal women (age range 42-80 years; mean age 58 years). Patients underwent oophorectomy for several different reasons. Two of the women reported a family history of breast and/or ovarian cancer, three of the women reported post-menopausal bleeding and all were diagnosed with endometrial cancer. In all cases, specimens were taken from normal appearing ovarian surface epithelium, which was confirmed following histopathological review. Two cases were found to contain a benign ovarian cyst upon review. Ovarian tissue specimens were removed either by laparotomy or laparoscopy. The ovarian surface epithelium was scraped from the ovarian surface and the cells were collected by centrifugation at 2000xg for five min. The cell pellets were plated into 25 cm^2^ tissue culture flasks using media described in [2]. Flasks were incubated at 37˚C in 5% CO_2_ without disturbance for two days to allow the cells to colonize. The medium was changed two times per week until cell were confluent, after which cells were trypsinized and transferred to new tissue culture flasks using 0.025% trypsin/0.01% at a ratio of 1:4.

Ovarian cancer tissue samples (19 serous, four clear cell and 10 endometrioid) were all snap-frozen specimens corresponding to Stage III/IV epithelial ovarian cancer. Each specimen was confirmed to contain >70% malignant epithelial cellularity as assessed by pathologist review of an adjacent stained tissue section. Ovarian cancer specimens were obtained from the Pacific Ovarian Cancer Research Consortium Repository, with the exception of one clear cell cancer specimen that was collected at Cedars Sinai Medical Center. Specimens were collected under Institutional Review Board approved protocols.

**RNA extraction and miRNA library preparation**

RNA was isolated from normal primary HOSE cultures at passage 4-7. Cells were washed with PBS and lysed in 600 µl mirVana Lysis/Binding buffer (Ambion, Inc., Austin, TX). Tumor specimens weighing approximately 100 mg each were homogenized in a Qiagen Tissuelyser with 600 μl mirVana Lysis/Binding buffer. Total RNA was extracted from both sample types using the mirVana^TM^ miRNA isolation kit (Ambion) per the manufacturer’s protocol. RNA was isolated from snap-frozen tumors (19 serous, four clear cell and 10 endometrioid tumors, respectively) and pooled according to histologic subtype. MicroRNA cloning was performed as previously described [3], <http://web.wi.mit.edu/bartel/pub/protocols/miRNACloningUpdate0705.pdf>) with modifications at the amplification steps to prepare the samples for massively parallel sequencing by 454 Life Sciences. Briefly, total RNA (ranging from 121 µg to 238 µg, depending on the sample) was combined with 25 fmol of 5´-^32^P-labeled 18 nt (AGCGUGUAGGGAUCCAAA) and 24 nt (GGCCAACGUUCUCAACAAUAGUGA) RNA molecular weight markers. Samples were then electrophoresed through 15% denaturing polyacrylamide gels (SequaGel Sequencing System, National Diagnostics, Atlanta, Georgia, USA). Markers were visualized by phosphorimaging and gel fragments containing the 18-24 nt fractions were excised with a scalpel. RNAs were eluted in 300 µl 0.3M NaCl with shaking at room temperature overnight, polyacrylamide was removed from the eluate using 0.2 µm Nanosep columns and the eluate was ethanol-precipitated with 20 µg of glycogen as a carrier. Precipitated RNAs were resuspended in DEPC-treated water and ligated at their 3’ ends with the DNA-based 3’ adaptor oligonucleotide, AppCTGTAGGCACCATCAATddC (Integrated DNA Technologies (IDT), Coralville, IA, USA) using T4 RNA ligase (Amersham Pharmacia Biotech (GE Healthcare Bio-Sciences), Piscataway, NJ, USA) in a 20 µl reaction at room temperature for two hours. 3’-ligated reaction products were resolved on a 12% denaturing polyacrylamide gel, imaged, excised, eluted and precipitated as above. A second ligation was then performed with the 5´-adaptor oligonucleotide, ATCGTaggcaccugaaa (Dharmacon, Inc., Chicago, IL, USA; uppercase, DNA; lowercase RNA), using T4 RNA ligase and ATP (0.4 mM) at 37^o^C for one hour. 5’-,3’-ligated products were purified through 10% denaturing polyacrylamide gels, imaged, excised, eluted and precipitated as described above. One third of the gel-purified 5’-,3’-ligated RNA was reverse-transcribed in a 30 µl reaction (SuperScript III, Invitrogen) using the RT primer ATTGATGGTGCCTAC. The cDNA was amplified by PCR (20 cycles), using AmpliTaq DNA polymerase (Applied Biosystems, Inc., Foster City, CA, USA), the RT primer and a DNA version of the 5´-adaptor oligonucleotide (5’PhosATCGTAGGCACCTGAGA). The PCR products (approximately 50 bp) were gel purified through 12% polyacrylamide, stained with Sybr Gold (Invitrogen), excised, eluted and precipitated as previously described. The products were resuspended in 20 ul water and 1 μl of the purified product was used as template for a second round of PCR using fusion primers to adapt the sequence for 454 sequencing (5’ primer: GCCTCCCTCGCGCCATCAGATCGTAGGCACCTGAGA, 3’ primer: GCCTTGCCAGCCCGCTCAGATTGATGGTGCCTACAG). Samples were amplified by AmpliTaq as follows: initial denaturation at 96^o^C for 60s, then (96^o^C for 10s, 50^o^C for 60s, 72^o^C for 20s) x 22 cycles, then 72^o^C extension for three min. Approximately 100 bp PCR products were gel purified through three percent metaphor agarose for two hours at 110V and excised using a clean scalpel. PCR products were recovered with the QiaQuick Gel Extraction Kit (Qiagen) using the manufacturer’s recommended protocol. Samples were eluted with 50 µl elution buffer. Samples were quantified by both Nanodrop spectrophotometer and PicoGreen assay (Invitrogen). The libraries were prepared for 454 sequencing (bead capture and emulsion PCR) using reagents and protocols recommended by 454 Life Sciences. The bead-captured, clonally amplified libraries were submitted for massively parallel pyrosequencing (454 Life Sciences, Inc., Branford, CT) [4].

**Custom bioinformatic data analysis pipeline**

The sequencing data was preprocessed by first trimming the 454 adaptor and 5’/3’ linker sequences from each read, followed by removal of any sequences corresponding to spiked-in RNA size markers (refer to **Figure S1**). Detailed information on the computational pipeline can be found in the Supplemental Methods of [5] with an abridged description below.

The data analysis pipeline identifies sequences that match previously annotated features in the genome and sets those sequences aside for further analysis. We first compared our sequences using BLAST [6] against a database constructed of known miRNA mature sequences (**Figure S1-a**) and a database constructed of the precursor sequences (miRBase release 12.0) [7,8] and then profiled the previously annotated miRNAs (**Figures 2, 3**).

Once we removed all the matches to known miRNAs, we then compared the sequences to other non-coding RNAs (**Figure S1-b)** including tRNAs, rRNAs, and piRNAs. Matches to the Repbase database were also removed (Dec. 2006 version) [9]. Exact matches to known genes in the RefSeq mRNA database (downloaded from the UCSC Table Browser) [10] (**Figure S1-c**) were also filtered out.

Once the annotated RNAs were removed, we BLASTed the remaining sequences against the human genome (UCSC Genome Browser, version hg18, March 2006 assembly, NCBI Build 36.1) (**Figure S1-d**). If a sequence mapped to more than 20 loci in the genome, it was removed from further analysis. When BLASTed against the human genome, we found that many sequences were not exact matches, with mismatches typically occurring at the 3’ end due to non-templated addition of 1-3 nucleotides. We chose to trim non-matching sequences if they had an exact match to the human genome starting at the 5’ end of the sequence and has a contiguous match of 17 or more nucleotides. The 3’ end of the sequence was trimmed back until the sequence was an exact match to the genome. The trimmed sequences were then re-screened for matches to annotated features in the genome as previously described.

For each genomic locus matching a particular small RNA sequence, we then extracted that sequence plus 100 nucleotides of up- and down-stream flanking sequence. All the extracted genomic sequences were then folded and filtered based on four criteria: free energy, shape probability, percent of base-pairing of the “mature” sequence within the hairpin and Randfold score (**Figure S1-e**). Free energy and shape probability were computed with the RNAshapes package [11]. Using the secondary structure given by RNAshapes, we calculated the percent base-pairing in the mature sequence part of the hairpin. The sequences with the best free energy and best shape probability were then given to the program Randfold [12]. We retained putative precursor sequences only where they met four criteria for appropriate folding: (a) the free energy was less than -34.0 *and* (b) the shape probability was greater than 90% *and* (c) the percent of base-pairing in the cloned sequence was greater than 70% *and* (d) the *P*-value given by Randfold was less than 0.08. We also required the mature sequence to appear in the stem of the hairpin shape.

Due to sequence variability at the 5' and 3' ends, many of these sequences arose from the same genomic locus and were super- or sub-sequences of each other or overlapped significantly. To choose a single “canonical sequence” from the multiple sequences arising from a single locus, we grouped the sequences by sorting with respect to the genomic coordinate of the 5’ end. In the sequence groups that shared a 5’ end, we selected the representative group for that locus based on which 5’ end group contained the more abundant sequence. We then summed the reads for the sequences within the 5’ group as the abundance of the canonical sequence. Any sequences less than 20 nts or greater than 24 nts were filtered out.

To compile additional supporting evidence for our hairpin structures, we assessed whether the structure was conserved across a phylogenetic range (**Figure S1-f**). Using the 17 vertebrate species alignment track from UCSC Genome Browser, homologous sequences which aligned to our mature sequences plus 100 basepairs up- and down-stream of the aligned sequence were extracted. We then applied the same folding criteria to these sequences to find hairpin orthologs (with the exception of base-pairing of the mature sequence since the mature sequence in the other species is unknown).

Additionally, we sought to identify sequences that shared seed region identity with known human miRNAs or known miRNAs from other species. The seed region of a miRNA is defined as nucleotides 2 through 8 in the mature sequence; this sequence is thought to determine mRNA targeting specificity [13]. We defined two sequences to have the same seed region if nucleotides 2–8 were identical.

To determine which of our canonical sequences were indeed novel miRNAs, we constructed a set of rules based on a combination of criteria involving abundance, membership in a 5’-3’ pair of a single hairpin structure, and shared seed region with known miRNAs. First, if a sequence was a member of a 5’-3’ hairpin pair where one of the sequences could be designated as “dominant” based on abundance of 3 or more reads, we considered it to be a valid novel miRNA. The less abundant sequence of the pair was annotated as the star form of the novel miRNA. Second, if a sequence was abundant (defined as represented by three or more reads) and it shared a seed region (nucleotides 2-8 of the mature sequence) with a known human miRNA, it was added to the list of novel miRNAs (**Figure S1-f**). Application of these rules gave us a total of six novel miRNAs, plus three star forms of novel miRNAs. To construct our list of “candidate miRNAs” from the remaining hairpin-derived small RNAs, we removed sequences that had two reads and no other supporting evidence (a shared seed region with known animal miRNA) or just a singleton read (**Figure S1-g**). This gave us a final list of 39 candidate miRNAs representing 50 genomic loci (**Table S4**).

**Measurement of miRNA levels in RNA from clinical samples using TaqMan qRT-PCR assays**

RNA (1.67 μl of total RNA (at 2.0 ng/μl) from pooled samples for each tumor subtype) was reverse transcribed using the TaqMan miRNA Reverse Transcription Kit and miRNA-specific stem-loop primers (Applied BioSystems, Inc.) in a small-scale RT reaction (comprised of 1.387 µl H_2_O, 0.5 µl 10X Reverse-Transcription Buffer, 0.063 µl RNase-Inhibitor (20 U/µl), 0.05 µl 100 mM dNTPs with dTTP, 0.33 µl Multiscribe™ Reverse-Transcriptase, 1.0 μl miRNA-specific stem-loop primers and 1.67 µl input RNA; components other than the primers and input RNA were prepared as a larger volume master mix), using a Tetrad2 Peltier Thermal Cycler (BioRad) at 16^o^C for 30 min, 42^o^C for 30 min and 85^o^C for 5 min. 2.25 µl of diluted RT product (prepared by combining 5.0 µl RT product with 28.9 µl H_2_O) was combined with 2.75 µl of a PCR assay reagents (comprised of 2.5 µl TaqMan 2X Universal PCR Master Mix, No AmpErase UNG and 0.25 µl TaqMan miRNA Assay) to generate a PCR reaction of 5.0 µl total volume. Real-time PCR was carried out on an Applied BioSystems 7900HT thermocycler (Applied Biosystems, Inc.) at 95°C for 10 min, followed by 40 cycles of 95°C for 15 s and 60°C for one min. Data was analyzed with SDS Relative Quantification Software version 2.2.2 (Applied BioSystems, Inc.), with the automatic Ct setting for assigning baseline and threshold for Ct determination.

Detailed information regarding specific miRNA TaqMan assays^†^ used in this study is provided below:

| **microRNA** | **ABI Part No.** | **Target Sequence** |
| --- | --- | --- |
| *hsa-miR-100* | 4373160 | AACCCGUAGAUCCGAACUUGUG |
| *hsa-miR-126** | 4373269 | CAUUAUUACUUUUGGUACGCG |
| *hsa-miR-127* | 4373147 | UCGGAUCCGUCUGAGCUUGGCU |
| *hsa-miR-141* | 4373137 | UAACACUGUCUGGUAAAGAUGG |
| *hsa-miR-142-3p* | 4373136 | UGUAGUGUUUCCUACUUUAUGGA |
| *hsa-miR-142-5p* | 4395359 | CAUAAAGUAGAAAGCACUACU |
| *hsa-miR-182* | 4373271 | UUUGGCAAUGGUAGAACUCACA |
| *hsa-miR-183* | 4395380 | UAUGGCACUGGUAGAAUUCACU |
| *hsa-miR-195* | 4373105 | UAGCAGCACAGAAAUAUUGGC |
| *hsa-miR-196a* | 4373104 | UAGGUAGUUUCAUGUUGUUGG |
| *hsa-miR-196b* | 4395326 | UAGGUAGUUUCCUGUUGUUGGG |
| *hsa-miR-200a* | 4378069 | UAACACUGUCUGGUAACGAUGU |
| *hsa-miR-200b* | 4395362 | UAAUACUGCCUGGUAAUGAUGA |
| *hsa-miR-200c* | 4395411 | UAAUACUGCCGGGUAAUGAUGGA |
| *hsa-miR-210* | 4373089 | CUGUGCGUGUGACAGCGGCUGA |
| *hsa-miR-22* | 4373079 | AAGCUGCCAGUUGAAGAACUGU |
| *hsa-miR-222* | 4395387 | AGCUACAUCUGGCUACUGGGU |
| *hsa-miR-375* | 4373027 | UUUGUUCGUUCGGCUCGCGUGA |
| *hsa-miR-378* | 4395354 | ACUGGACUUGGAGUCAGAAGG |
| *hsa-miR-382* | 4373019 | GAAGUUGUUCGUGGUGGAUUCG |
| *hsa-miR-409-5p* | 4395442 | AGGUUACCCGAGCAACUUUGCAU |
| *hsa-miR-429* | 4373203 | UAAUACUGUCUGGUAAAACCGU |
| *hsa-miR-449* | 4373207 | UGGCAGUGUAUUGUUAGCUGGU |
| *hsa-miR-485-5p* | 4373212 | AGAGGCUGGCCGUGAUGAAUUC |
| *hsa-miR-493-3p* | 4378100 | UGAAGGUCUACUGUGUGCCAG |
| *hsa-miR-9* | 4373285 | UCUUUGGUUAUCUAGCUGUAUGA |
| *hsa-miR-96* | 4373010 | UUUGGCACUAGCACAUUUUUGC |

^†^miRNA names and sequences are based on ABI product specifications, and do not necessarily correlate to current version of miRBase.

**Supplementary Figure Legend**

**Figure S1. Flow chart of sequence data analysis pipeline**. The flow chart shows the steps in the computational analysis of the 454 sequencing data. At each step, sequences may be removed for further analysis, or carried on to the next step in the pipeline. The first steps remove previously annotated features from the pipeline, and then remaining sequences are tested for presence of hairpin secondary structure and other criteria to be designated novel miRNAs. Bolded lower-case letters are referred to in the **Supplementary Methods.**

**Supplemental References**

1. Dyck HG, Hamilton TC, Godwin AK, Lynch HT, Maines-Bandiera S, et al. (1996) Autonomy of the epithelial phenotype in human ovarian surface epithelium: changes with neoplastic progression and with a family history of ovarian cancer. Int J Cancer 69: 429-436.

2. Li NF, Wilbanks G, Balkwill F, Jacobs IJ, Dafou D, et al. (2004) A modified medium that significantly improves the growth of human normal ovarian surface epithelial (OSE) cells in vitro. Lab Invest 84: 923-931.

3. Lau NC, Lim LP, Weinstein EG, Bartel DP (2001) An abundant class of tiny RNAs with probable regulatory roles in Caenorhabditis elegans. Science 294: 858-862.

4. Margulies M, Egholm M, Altman WE, Attiya S, Bader JS, et al. (2005) Genome sequencing in microfabricated high-density picolitre reactors. Nature 437: 376-380.

5. Bar M, Wyman SK, Fritz BR, Qi J, Garg KS, et al. (2008) MicroRNA Discovery and Profiling in Human Embryonic Stem Cells by Deep Sequencing of Small RNA Libraries. Stem Cells.

6. Altschul SF, Gish W, Miller W, Myers EW, Lipman DJ (1990) Basic local alignment search tool. J Mol Biol 215: 403-410.

7. Griffiths-Jones S, Grocock RJ, van Dongen S, Bateman A, Enright AJ (2006) miRBase: microRNA sequences, targets and gene nomenclature. Nucleic Acids Res 34: D140-144.

8. Griffiths-Jones S (2004) The microRNA Registry. Nucleic Acids Res 32: D109-111.

9. Jurka J (2000) Repbase update: a database and an electronic journal of repetitive elements. Trends Genet 16: 418-420.

10. Kent WJ, Sugnet CW, Furey TS, Roskin KM, Pringle TH, et al. (2002) The human genome browser at UCSC. Genome Res 12: 996-1006.

11. Steffen P, Voss B, Rehmsmeier M, Reeder J, Giegerich R (2006) RNAshapes: an integrated RNA analysis package based on abstract shapes. Bioinformatics 22: 500-503.

12. Bonnet E, Wuyts J, Rouze P, Van de Peer Y (2004) Evidence that microRNA precursors, unlike other non-coding RNAs, have lower folding free energies than random sequences. Bioinformatics 20: 2911-2917.

13. Lewis BP, Burge CB, Bartel DP (2005) Conserved seed pairing, often flanked by adenosines, indicates that thousands of human genes are microRNA targets. Cell 120: 15-20.
