# Supplementary figures and images for: Repertoire of microRNAs in Epithelial Ovarian Cancer as Determined by Next Generation Sequencing of Small RNA cDNA Libraries
Source: PLoS One. 2009 Apr 23;4(4):e5311. doi: 10.1371/journal.pone.0005311 (PMC2668797; doi:10.1371/journal.pone.0005311)

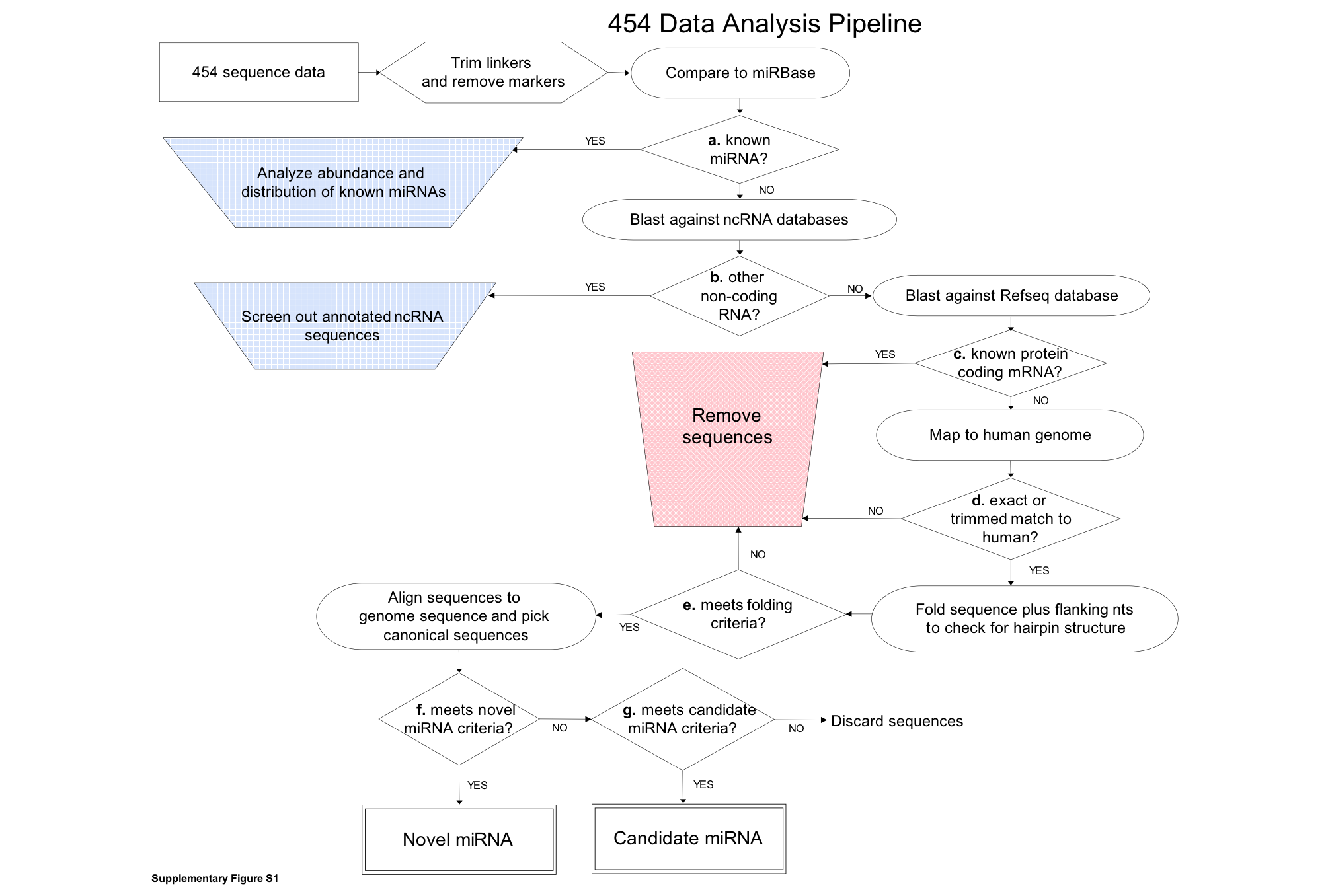

Supplement: Figure S1 — Flow chart of sequence data analysis pipeline. The flow chart shows the steps in the computational analysis of the 454 sequencing data. At each step, sequences may be removed for further analysis, or carried on to the next step in the pipeline. The first steps remove previously annotated features from the pipeline, and then remaining sequences are tested for presence of hairpin secondary structure and other criteria to be designated novel miRNAs. Bolded lower-case letters are referred to in the Supplementary Methods. (0.77 MB TIF) [file pone.0005311.s007.tif]
